# Supplementary material for: Biomarkers of the L-Arginine/Dimethylarginine/Nitric Oxide Pathway in People with Chronic Airflow Obstruction and Obstructive Sleep Apnoea
Source: J Clin Med. 2023 Aug 11;12(16):5230. doi: 10.3390/jcm12165230 (PMC10455103; doi:10.3390/jcm12165230)
Supplement: Supplementary file 1 [file jcm-12-05230-s001.zip › jcm-2516747-SI.pdf]

Biomarkers of the L-arginine / dimethylarginine / nitric oxide pathway in people with chronic airflow obstruction and obstructive sleep apnoea

Juliane Hannemann[1,2#]*, Elin H. Thorarinnsdottir[3,4#], André F.S. Amaral[5,6], Edzard Schwedhelm[1], Lena Schmidt-Hutten[1], Heike Stang[1], Bryndis Benediktsdottir[4,7], Ingibjörg Gunnarsdottir[8], Thorarinn Gislason[4,7#], Rainer Böger[1,2#]

SUPPLEMENTARY TABLES

[1]Institute of Clinical Pharmacology and Toxicology, University Medical Center Hamburg-Eppendorf, Hamburg, Germany; [2]Institute DECIPHER, German-Chilean Institute for Research on Pulmonary Hypoxia and its Health Sequelae, Hamburg, Germany; [3]Primary Health Care of the Capital Area, Reykjavik, Iceland; [4] Faculty of Medicine, University of Iceland, Reykjavik, Iceland; [5]National Heart and Lung Institute, Imperial College London, London, U.K.; [6]NIHR Imperial Biomedical Research Centre, London, U.K.; [7]Sleep Department, Landspitali University Hospital of Iceland, Reykjavik, Iceland; [8]Unit for Nutrition Research, Landspitali University Hospital & Faculty of Food Science and Nutrition, University of Iceland, Reykjavik, Iceland.

Table S1. Chronic airflow obstruction, respiratory symptoms, and sleep-related symptoms in the study population by sex.

Variable	Total	Female	Male	P*
**Chronic airflow obstruction[1]**				
Yes	78 (10.9)	46 (13.7)	32 (8.5)	**0.03**
**Respiratory symptoms[2]**				
Chronic cough	82 (11.5)	41 (12.2)	41 (10.9)	0.58
Chronic phlegm	67 (9.4)	28 (8.3)	39 (10.3)	0.36
Wheezing	173 (24.3)	79 (23.5)	94 (24.9)	0.66
**Sleepiness**				
Mean ESS score	6.1 ± 3.9	5.7 ± 3.9	6.4 ± 3.9	**0.017**
ESS > 10	94 (13.2)	40 (11.9)	54 (14.3)	0.34
Daytime sleepiness ≥ 3 days/week	147 (20.8)	74 (22.2)	73 (19.5)	0.38
Feeling rested < 1 time/week	99 (17.4)	46 (17.7)	53 (17.2)	0.88
**OSA-related symptoms**				
Loud snoring ≥ 3 nights/week	134 (26.0)	33 (15.1)	101 (34.0)	**<0.001**
Observed apneas ≥ 1 night/week	46 (8.7)	9 (3.6)	37 (13.2)	**<0.001**
Nocturnal sweating ≥ 3 nights/week	76 (10.8)	41 (12.4)	35 (9.4)	0.21
Nocturnal GER ≥ 1 night/week	40 (5.7)	21 (6.4)	19 (5.1)	0.46
**MAP Index**				
Mean MAP Index	0.36 ± 0.23	0.21 ± 0.16	0.50 ± 0.19	**<0.001**
MAP > 0.5	215 (30.2)	24 (7.1)	191 (50.7)	**<0.001**

Data are presented as mean ± standard deviation and as number of observations (n) with the percent value given in brackets. *p values from chi-squared test (categorical) and Student's t test (continuous) comparing males with females. [1]Chronic airflow obstruction was defined as post-bronchodilator FEV1/FVC < lower limit of the normal range as described by [7]. [2]Chronic cough or phlegm was defined as positive if present for three months or more per year, wheezing was defined as positive if wheezing / whistling in the chest had been noted at any time during the preceding 12 months. Abbreviations: ESS, Epworth Sleepiness Scale; nGER, nocturnal gastroesophageal reflux; MAP, Multivariable Apnea Prediction Index; OSA, obstructive sleep apnea.

Table S2. Distribution of biomarkers of interest by age group and smoking status.

Variable	Age (years)					Smoking status			
	**40-49** (n=232)	**50-59** (n=220)	**60-69** (n=139)	**≥70** (n=122)	**p-value***	**Never** (n=278)	**Past** (n=302)	**Current** (n=131)	**p-value***
ADMA, median (IQR)	0.48 (0.42, 0.54)	0.50 (0.45, 0.55)	0.51 (0.46, 0.56)	0.54 (0.49, 0.59)	**<0.001**	0.50 (0.44, 0.55)	0.51 (0.45, 0.57)	0.52 (0.46, 0.57)	0.16
SDMA, median (IQR)	0.47 (0.42, 0.54)	0.48 (0.44, 0.54)	0.51 (0.46, 0.60)	0.60 (0.51, 0.69)	**<0.001**	0.50 (0.45, 0.58)	0.50 (0.45, 0.58)	0.46 (0.42, 0.54)	**0.003**
L-Arginine, median (IQR)	137.2 (125.3, 158.6)	149.9 (130.4, 164.5)	146.2 (127.9, 166.5)	140.7 (126.4, 162.0)	**0.008**	138.0 (123.9, 158.2)	146.2 (129.0, 165.2)	149.4 (134.5, 169.2)	**<0.001**
L-Citrulline, median (IQR)	36.6 (30.9, 43.3)	38.9 (32.5, 45.7)	41.8 (36.0, 49.6)	45.8 (38.4, 56.8)	**<0.001**	39.7 (33.6, 47.3)	39.7 (32.3, 48.0)	39.0 (32.8, 46.7)	0.97
L-Ornithine, median (IQR)	75.9 (62.1, 91.0)	79.7 (68.4, 93.4)	81.0 (66.8, 96.7)	86.3 (75.5, 102.6)	**<0.001**	79.1 (66.7, 92.5)	80.9 (67.3, 95.3)	81.4 (67.3, 99.5)	0.38
Arg/ADMA Ratio, median (IQR)	296.3 (252.6, 330.5)	294.8 (262.6, 345.1)	287.9 (250.2, 332.4)	269.4 (240.8, 299.4)	**<0.001**	284.6 (248.6, 323.8)	290.2 (255.4, 339.1)	290.7 (259.0, 326.7)	0.11
Cit/Arg Ratio, median (IQR)	0.26 (0.22, 0.31)	0.26 (0.22, 0.32)	0.28 (0.23, 0.34)	0.32 (0.26, 0.40)	**<0.001**	0.28 (0.24, 0.34)	0.27 (0.22, 0.33)	0.26 (0.22, 0.31)	0.05
Orn/Arg Ratio, median (IQR)	0.52 (0.44, 0.66)	0.54 (0.45, 0.65)	0.55 (0.46, 0.65)	0.60 (0.52, 0.70)	**0.0001**	0.57 (0.46, 0.67)	0.54 (0.46, 0.66)	0.53 (0.45, 0.65)	0.36

Data are presented as medians and inter quartile ranges. *p-value from Kruskal-Wallis test. Abbreviations: ADMA, asymmetric dimethylarginine; Arg, L-arginine; BMI, body mass index; Cit, L-citrulline; Orn, L-ornithine; SDMA, symmetric dimethylarginine.

Table S3. Chronic airflow obstruction and/or obstructive sleep apnoea risk and biomarker concentrations among the study population stratified by BMI categories.

**Variable**	**<20**	**20.0-22.5**	**22.5-25**	**25-27.5**	**27.5-30**	**30-32.5**	**32.5-35**	**>35**	**p-value***
N (%)	13 (1.8)	57 (8.0)	131 (18.4)	167 (23.4)	145 (20.3)	97 (13.6)	50 (7.0)	53 (7.4)	-
Males (%)	4 (30.8)	18 (31.6)	57 (43.5)	95 (56.9)	93 (64.1)	58 (59.8)	29 (58.0)	23 (43.4)	**<0.001**
Age (years)	65 ± 13	55 ± 12	55 ± 11	58 ± 13	58 ± 12	57 ± 11	61 ± 11	55 ± 9	**0.006**
**MAP/CAO categories**									
-MAP/-CAO	7 (53.9)	47 (82.5)	100 (76.3)	122 (73.1)	91 (62.8)	45 (46.4)	16 (32.0)	11 (20.8)	**<0.001**
-MAP/+CAO	6 (46.2)	9 (15.7)	19 (14.5)	14 (8.4)	5 (3.5)	2 (2.1)	3 (6.0)	1 (1.9)	
+MAP/-CAO	0 (0)	0 (0)	11 (8.4)	26 (15.6)	44 (30.4)	48 (49.5)	28 (56.0)	39 (73.6)	
+MAP/+CAO	0 (0)	1 (1.8)	1 (0.8)	5 (3.0)	5 (3.5)	2 (2.1)	3 (6.0)	2 (3.8)	
**Biomarker levels**									
ADMA (µmol/L)	0.52 (0.49, 0.58)	0.48 (0.45, 0.55)	0.51 (0.43, 0.57)	0.50 (0.44, 0.57)	0.50 (0.45, 0.55)	0.50 (0.44, 0.54)	0.52 (0.47, 0.55)	0.53 (0.48, 0.59)	0.092
SDMA (µmol/L)	0.54 (0.48, 0.63)	0.49 (0.44, 0.55)	0.49 (0.45, 0.57)	0.52 (0.45, 0.60)	0.50 (0.45, 0.58)	0.51 (0.44, 0.55)	0.49 (0.44, 0.61)	0.47 (0.44, 0.50)	**0.025**
L-Arginine (µmol/L)	142.9 (125.6, 159.7)	135.9 (124.1, 158.0)	141.0 (123.5, 163.2)	142.5 (125.9, 164.1)	144.0 (132.3, 163.1)	150.1 (129.6, 167.6)	147.1 (134.9, 161.2)	147.8 (128.6, 178.8)	0.336
L-Citrulline (µmol/L)	39.1 (31.4, 54.8)	37.7 (31.6, 46.3)	39.8 (32.9, 47.2)	39.2 (34.3, 47.2)	40.7 (33.9, 50.0)	37.4 (31.1, 44.4)	44.1 (33.3, 54.0)	39.3 (33.6, 48.4)	0.089
L-Ornithine (µmol/L)	81.4 (61.6, 89.9)	70.9 (61.3, 88.4)	78.9 (65.9, 93.6)	85.0 (70.7, 98.1)	79.7 (67.4, 95.4)	76.8 (66.8, 91.5)	83.8 (66.4, 95.3)	85.5 (75.7, 102.0)	**0.004**
Arg / ADMA Ratio	275.0 (236.5, 295.9)	282.8 (253.7, 312.9)	293.8 (247.3, 334.6)	277.1 (246.2, 323.8)	295.1 (259.5, 333.2)	303.3 (264.5, 335.7)	294.1 (259.7, 328.7)	281.3 (244.0, 330.2)	0.128
Cit / Arg Ratio	0.26 (0.23, 0.40)	0.28 (0.23, 0.35)	0.27 (0.23, 0.33)	0.29 (0.24, 0.33)	0.28 (0.23, 0.33)	0.24 (0.21,0.30)	0.30 (0.22,0.38)	0.27 (0.23, 0.32)	**0.016**
Orn / Arg Ratio	0.55 (0.47, 0.60)	0.53 (0.46, 0.64)	0.55 (0.44, 0.66)	0.58 (49, 0.71)	0.53 (0.46, 0.66)	0.50 (0.44, 0.62)	0.56 (0.47, 0.67)	0.59 (0.49, 0.74)	**0.012**

Data are presented as mean ± standard deviation or as median with interquartile ranges in square brackets for continuous variables as appropriate. Categorical variables are presented as number of observations (N) with the percent value given in brackets. *p values from Student's t test for means, Kruskal-Wallis test for medians (continuous) and Chi-square test (categorical). Abbreviations: ADMA, asymmetric dimethylarginine; Arg, L-arginine; BMI, body mass index; CAO, Chronic airflow obstruction, Cit, L-citrulline; CVD, cardiovascular disease; MAP, Multivariable apnea prediction index, Orn, L-ornithine; SDMA, symmetric dimethylarginine.

Table S4. Distribution of biomarkers of interest based on the presence or absence of hypertension, cardiovascular disease and diabetes.

	**Hypertension**			**Cardiovascular disease**			**Diabetes**		
	**Yes** (n=177)	**No** (n=524)	**p-value***	**Yes** (n=106)	**No** (n=606)	**p-value***	**Yes** (n=20)	**No** (n=689)	**p-value***
ADMA, median (IQR)	0.52 (0.46, 0.58)	0.50 (0.44, 0.55)	**0.02**	0.50 (0.46, 0.58)	0.51 (0.44, 0.56)	0.24	0.51 (0.43, 0.61)	0.51 (0.45, 0.56)	0.79
SDMA, median (IQR)	0.53 (0.46, 0.62)	0.49 (0.44, 0.56)	**<0.001**	0.54 (0.49, 0.64)	0.49 (0.44, 0.56)	**<0.001**	0.51 (0.47, 0.63)	0.50 (0.44, 0.57)	0.08
L-Arginine, median (IQR)	144.5 (130.0, 164.0)	143.3 (126.3, 162.8)	0.42	142.9 (125.3, 164.1)	144.2 (127.7, 163.2)	0.28	140.0 (129.4, 156.8)	144.4 (126.6, 163.2)	0.82
L-Citrulline, median (IQR)	42.8 (35.8, 52.0)	38.4 (32.0, 46.1)	**<0.001**	45.2 (36.0, 54.3)	39.0(32.5, 46.1)	**<0.001**	29.2 (22.0, 43.5)	39.6 (33.4, 47.4)	**0.01**
L-Ornithine, median (IQR)	83.5 (67.3, 96.4)	79.8 (66.8, 94.7)	0.29	85.5 (67.1, 101.2)	79.7 (66.8, 94.2)	0.11	83.9 (769.0, 87.8)	80.3 (66.8, 95.3)	0.95
Arg/ADMA Ratio, median (IQR)	285.0 (252.3, 327.8)	289.5(251.3, 333.5)	0.40	284.6 (240.8, 321.1)	289.7 (253.4, 333.4)	0.08	284.6 (250.3, 315.2)	288.7 (251.3, 330.2)	0.50
Cit/Arg Ratio, median (IQR)	0.29 (0.24, 0.35)	0.27 (0.22, 0.32)	**<0.001**	0.30 (0.25, 0.38)	0.27 (0.22, 0.32)	**<0.001**	0.22 (0.17, 0.30)	0.27 (0.23, 0.33)	**0.01**
Orn/Arg Ratio, median (IQR)	0.55 (0.47, 0.68)	0.55 (0.46, 0.66)	0.57	0.58 (0.49, 0.68)	0.55 (0.45, 0.66)	**0.03**	0.56 (0.49, 0.66)	0.55 (0.46, 0.67)	0.79

Data are presented as medians and inter quartile ranges. *p-value from Kruskal-Wallis test. Abbreviations: ADMA, asymmetric dimethylarginine; Arg, L-arginine; Cit, L-citrulline; Orn, L-ornithine; SDMA, symmetric dimethylarginine.

Table S5. Association of chronic airflow obstruction with biomarkers of interest. Model 1: Unadjusted. Model 2: Adjusted for age, sex and smoking status. Model 3: Model 2+BMI. Model 4: Model 2+eGFR.

**ADMA quartile**	**Concentration in uM**	**N**	**Model 1 OR (95% CI)**	**p**	**Model 2 OR (95% CI)**	**p**	**Model 3 OR (95% CI)**	**p**	**Model 4 OR (95% CI)**	**p**
1	0.2965-0.4475	179	1 (Ref)		1 (Ref)		1 (Ref)		1 (Ref)	
2	0.4480-0.5065	178	0.90 (0.45-1.77)	0.74	0.70 (0.34-1.43)	0.33	0.69 (0.34-1.42)	0.32	0.70 (0.34-1.42)	0.32
3	0.5065-0.5590	179	0.83 (0.41-1.66)	0.59	0.58 (0.28-1.21)	0.15	0.65 (0.31-1.36)	0.25	0.58 (0.28-1.21)	0.15
4	0.5600-1.1335	177	1.45 (0.77-2.73)	0.25	0.94 (0.48-1.84)	0.86	0.98 (0.50-1.94)	0.96	0.94 (0.48-1.84)	0.85
**SDMA quartile**	**Concentration in uM**									
1	0.2950-0.4425	180	1 (Ref)		1 (Ref)		1 (Ref)		1 (Ref)	
2	0.4435-0.4980	179	1.16 (0.55-2.46)	0.69	1.20 (0.56-2.59)	0.64	1.24 (0.57-2.70)	0.59	1.23 (0.57-2.66)	0.61
3	0.499-0.5740	176	1.10 (0.52-2.36)	0.80	1.10 (0.50-2.41)	0.82	1.05 (0.47-2.32)	0.91	1.13 (0.51-2.51)	0.76
4	0.5750-1.8515	178	2.70 (1.39-5.24)	**0.003**	2.10 (1.00-4.37)	**0.049**	1.99 (0.94-4.21)	0.07	2.24 (1.03-4.89)	**0.042**
**L-Arginine quartile**	**Concentration in uM**									
1	65.705-126.905	179	1 (Ref)		1 (Ref)		1 (Ref)		1 (Ref)	
2	126.958-144.036	178	0.70 (0.36-1.39)	0.31	0.63 (0.31-1.27)	0.19	0.68 (0.33-1.38)	0.28	0.63 (0.31-1.27)	0.19
3	144.072-163.145	178	0.85 (0.44-1.64)	0.63	0.77 (0.39-1.51)	0.44	0.84 (0.42-1.68)	0.63	0.77 (0.39-1.51)	0.44
4	163.273-279.045	178	0.95 (0.50-1.81)	0.89	0.81 (0.41-1.57)	0.53	0.92 (0.47-1.82)	0.82	0.80 (0.41-1.56)	0.51
**L-Citrulline quartile**	**Concentration in uM**									
1	15.515-33.009	179	1 (Ref)		1 (Ref)		1 (Ref)		1 (Ref)	
2	33.054-39.521	178	0.76 (0.37-1.59)	0.47	0.67 (0.32-1.41)	0.29	0.67 (0.29-1.33)	0.22	0.67 (0.32-1.42)	0.29
3	39.527-47.328	178	1.26 (0.65-2.44)	0.49	1.03 (0.52-2.05)	0.93	1.06 (0.53-2.12)	0.95	1.03 (0.51-2.04)	0.94
4	47-406-132.089	178	1.39 (0.73-2.67)	0.32	0.88 (0.43-1.77)	0.71	0.88 (0.43-1.80)	0.47	0.86 (0.42-1.76)	0.69
**L-Ornithine quartile**	**Concentration in uM**									
1	33.585-66.966	179	1 (Ref)		1 (Ref)		1 (Ref)		1 (Ref)	
2	67.066-80.412	178	0.72 (0.37-1.39)	0.33	0.59 (0.30-1.18)	0.14	0.63 (0.31-1.426)	0.19	0.59 (0.30-1.18)	0.14
3	80.436-95.332	178	0.72 (0.37-1.39)	0.33	0.57 (0.29-1.16)	0.12	0.63 (0.31-1.28)	0.20	0.57 (0.28-1.15)	0.12
4	95.339-280.306	178	0.91 (0.48-1.71)	0.76	0.62 (0.32-1.21)	0.16	0.68 (0.34-1.34)	0.27	0.62 (0.32-1.21)	0.16
**Arg/ADMA quartile**	**Ratio**									
1	74.2429-251.197	179	1 (Ref)		1 (Ref)		1 (Ref)		1 (Ref)	
2	251.326-288.691	178	0.68 (0.35-1.32)	0.26	0.60 (0.30-1.19)	0.14	0.55 (0.27-1.10)	0.09	0.60 (0.30-1.19)	0.14
3	288.877-330.143	178	0.64 (0.33-1.25)	0.19	0.66 (0.33-1.32)	0.24	0.68 (0.33-1.37)	0.28	0.66 (0.33-1.32)	0.24
4	330.197-532.619	178	0.86 (0.46-1.62)	0.65	1.02 (0.53-1.98)	0.94	1.01 (0.52-1.98)	0.97	1.02 (0.53-1.98)	0.95
**Cit/Arg quartile**	**Ratio**									
1	0.083-0.2268	179	1 (Ref)		1 (Ref)		1 (Ref)		1 (Ref)	
2	0.2274-0.2732	178	1.07 (0.53-2.15)	0.85	1.13 (0.55-2.30)	0.74	1.03 (0.50-2.12)	0.94	1.13 (0.55-2.32)	0.73
3	0.2734-0.3279	178	1.34 (0.69-2.63)	0.39	1.26 (0.63-2.53)	0.51	1.17 (0.58-2.37)	0.66	1.27 (0.63-2.55)	0.50
4	0.3291-0.8897	178	1.27 (0.65-2.51)	0.48	0.93 (0.45-1.91)	0.45	0.83 (0.40-1.72)	0.61	0.92 (0.45-1.89)	0.82
**Orn/Arg quartile**	**Ratio**									
1	0.2392-0.4580	179	1 (Ref)		1 (Ref)		1 (Ref)		1 (Ref)	
2	0.4582-0.5523	178	1.29 (0.64-2.58)	0.47	1.03 (0.50-2.11)	0.93	1.06 (0.52-2.18)	0.88	1.03 (0.51-2.12)	0.93
3	0.5529-0.6666	178	1.59 (0.81-3.10)	0.18	1.23 (0.61-2.47)	0.56	1.19 (0.59-2.42)	0.62	1.23 (0.61-2.48)	0.56
4	0.6678-1.8134	178	1.15 (0.56-2.33)	0.71	0.90 (0.43-1.89)	0.78	0.93 (0.44-1.97)	0.85	0.90 (0.43-1.89)	0.78

Data are presented as odds ratios and 95% confidence intervals with subjects without chronic airflow obstruction as reference. Abbreviations: ADMA, asymmetric dimethylarginine; Arg, L-arginine; CI, confidence interval; Cit, L-citrulline; OR, Odds ratio; Orn, L-ornithine; SDMA, symmetric dimethylarginine.

Table S6. Distribution of biomarkers of interest based on the presence or absence of sleepiness.

	**Epworth sleepiness scale**			**Feeling sleepy**			**Feeling rested**		
	**ESS score ≤10** (n=619)	**ESS score >10** (n=94)	**p-value***	**<3 times/week** (n=560)	**≥3 days/week** (n=147)	**p-value***	**>1 days/week** (n=469)	**<1 day/week** (n=99)	**p-value***
ADMA	0.51 (0.45, 0.56)	0.50 (0.45, 0.55)	0.567	0.50 (0.44, 0.55)	0.53 (0.46, 0.58)	**0.004**	0.51 (0.44, 0.56)	0.53 (0.46, 0.59)	0.052
SDMA	0.50 (0.44, 0.58)	0.50 (0.44, 0.54)	0.229	0.49 (0.44, 0.56)	0.51 (0.46, 0.60)	**0.007**	0.50 (0.44, 0.58)	0.51 (0.46, 0.60)	0.137
L-Arginine	143.5 (126.4, 163.5)	146.7 (131.9, 161.7)	0.338	144.5 (127.8, 162.5)	142.4 (126.1, 1667.0)	0.966	142.2 (126.1, 161.6)	150.7 (132.6, 172.9)	**0.031**
L-Citrulline	39.6 (33.3, 47.7)	39.1 (31.4, 46.7)	0.476	39.7 (32.9, 47.4)	38.9 (32.9, 48.4)	0.949	40.1 (33.3, 48.0)	39.4 (33.9, 48.4)	0.803
L-Ornithine	80.4 (66.8, 95.3)	80.5 (69.2, 95.0)	0.809	79.8 (66.8, 95.2)	81.4 (67.1, 95.5)	0.540	80.4 (67.3, 95.3)	81.4 (68.1, 97.2)	0.675
Arg/ADMA Ratio	287.1 (250.6, 328.8)	298.1 (255.9, 342.4)	0.251	289.7 (255.6, 333.8)	280.0 (240.0, 324.6)	0.052	288.3 (250.4, 325.8)	289.0 (250.9, 329.6)	0.634
Cit/Arg Ratio	0.27 (0.23, 0.33)	0.27 (0.22, 0.32)	0.218	0.27 (0.23, 0.33)	0.27 (0.23, 0.33)	0.921	0.28 (0.23, 0.33)	0.26 (0.22, 0.33)	0.254
Cit/Orn Ratio	0.515 (0.46, 0.67)	0.55 (0.45, 0.66)	0.473	0.55 (0.46, 0.67)	0.56 (0.46, 0.66)	0.687	0.56 (0.47, 0.67)	0.54 (0.45, 0.67)	0.374

Data are presented as medians and inter quartile ranges. *p-value from Kruskal-Wallis test. Abbreviations: ADMA, asymmetric dimethylarginine; Arg, L-arginine; Cit, L-citrulline; ESS, Epworth sleepiness scale; Orn, L-ornithine; SDMA, symmetric dimethylarginine.

Table S7. Association of high obstructive sleep apnoea risk as assessed by the MAP index **≥ 0.5** with quartiles of biomarkers of interest.
Model 1: Unadjusted. Model 2: Adjusted for eGFR

**ADMA quartile**	**Concentration (µmol/L)**	**N**	**Model 1 OR (95% CI)**	**p-value**	**Model 2 OR (95% CI)**	**p-value**
1	0.2965-0.4475	179	1 (Ref)		1 (Ref)	
2	0.4480-0.5065	178	1.07 (0.66, 1.72)	0.785	1.06 (0.66, 1.71)	0.806
3	0.5065-0.5590	179	1.50 (0.95, 2.38)	0.082	1.49 (0.94, 2.36)	0.089
4	0.5600-1.1335	177	1.65 (1.04, 2.60)	**0.033**	1.63 (1.03, 2.58)	**0.037**
**SDMA quartile**	**Concentration (µmol/L)**					
1	0.2950-0.4425	180	1 (Ref)		1 (Ref)	
2	0.4435-0.4980	179	1.54 (0.96, 2.47)	0.070	1.57 (0.98, 2.51)	0.063
3	0.499-0.5740	176	1.35 (0.83, 2.17)	0.224	1.38 (0.85, 2.25)	0.190
4	0.5750-1.8515	178	2.10 (1.32, 3.32)	**0.002**	2.23 (1.35, 3.69)	**0.002**
**L-Arginine quartile**	**Concentration (µmol/L)**					
1	65.705-126.905	179	1 (Ref)		1 (Ref)	
2	126.958-144.036	178	1.13 (0.71, 1.80)	0.612	1.13 (0.71, 1.80)	0.609
3	144.072-163.145	178	1.32 (0.84, 2.09)	0.231	1.33 (0.84, 2.10)	0.228
4	163.273-279.045	178	1.43 (0.91, 2.25)	0.125	1.342 (0.90, 2.24)	0.132
**L-Citrulline quartile**	**Concentration (µmol/L)**					
1	15.515-33.009	179	1 (Ref)		1 (Ref)	
2	33.054-39.521	178	1.10 (0.67, 1.74)	0.698	1.10 (0.69, 1.74)	0.701
3	39.527-47.328	178	1.10 (0.69, 1.74)	0.698	1.09 (0.69, 1.74)	0.707
4	47-406-132.089	178	1.57 (1.00, 2.46)	**0.049**	1.56 (0.98, 2.48)	0.059
**L-Ornithine quartile**	**Concentration (µmol/L)**					
1	33.585-66.966	179	1 (Ref)		1 (Ref)	
2	67.066-80.412	178	1.13 (0.70, 1.83)	0.604	1.13 (0.70, 1.82)	0.615
3	80.436-95.332	178	1.90 (1.21, 3.01)	**0.006**	1.90 (1.23, 3.00)	**0.006**
4	95.339-280.306	178	1.53 (0.96, 2.43)	0.073	1.52 (0.95, 2.42)	0.079
**Arg/ADMA quartile**	**Ratio**					
1	74.2429-251.197	179	1 (Ref)		1 (Ref)	
2	251.326-288.691	178	0.50 (0.32, 0.81)	**0.004**	0.50 (0.32, 0.81)	**0.004**
3	288.877-330.143	178	0.82 (0.53, 1.28)	0.391	0.82 (0.53, 1.28)	0.388

4	330.197-532.619	178	0.82 (0.53, 1.28)	0.391	0.83 (0.53, 1.29)	0.402
**Cit/Arg quartile**	**Ratio**					
1	0.083-0.2268	179	1 (Ref)		1 (Ref)	
2	0.2274-0.2732	178	0.47 (0.28, 0.75)	**0.002**	0.47 (0.29, 0.75)	**0.002**
3	0.2734-0.3279	178	0.94 (0.60, 1.44)	0.605	0.93 (0.60, 1.45)	0.760
4	0.3291-0.8897	178	0.85 (0.54, 1.31)	0.544	0.83 (0.53, 1.30)	0.414
**Orn/Arg quartile**	**Ratio**					
1	0.2392-0.4580	179	1 (Ref)		1 (Ref)	
2	0.4582-0.5523	178	1.53 (0.96, 2.43)	0.073	1.52 (0.96, 2.42)	0.076
3	0.5529-0.6666	178	1.65 (1.04, 2.62)	**0.034**	1.64 (1.03, 2.60)	**0.037**
4	0.6678-1.8134	178	1.34 (0.84, 2.15)	0.221	1.33 (0.83, 2.14)	0.229

Data are presented as odds ratios and 95% confidence intervals with subjects MAP index <0.5 as reference. Abbreviations: ADMA, asymmetric dimethylarginine; Arg, L-arginine; CI, confidence interval; Cit, L-citrulline; OR, Odds ratio; Orn, L-ornithine; SDMA, symmetric dimethylarginine.
